# Scoping review on mental health standards for Black youth: identifying gaps and promoting equity in community, primary care, and educational settings

**DOI:** 10.1186/s13034-024-00800-5

**Published:** 2024-09-09

**Authors:** Ruth Martínez-Vega, Aloysius Nwabugo Maduforo, Andre Renzaho, Dominic A. Alaazi, Dzifa Dordunoo, Modupe Tunde-Byass, Olutoyosi Unachukwu, Victoria Fajenyo, Alicia Boatswain-Kyte, Geoffrey Maina, Barbara-Ann Hamilton-Hinch, Notisha Massaquoi, Azeez Salami, Oluwabukola Salami

**Affiliations:** 1 Department of Community Health Sciences, Cumming School of Medicine, University of Calgary, 3280 Hospital Drive, Calgary, Canada; 2 School of Medicine, Western Sydney University, David Pilgrim AvenueNSW2560, Campbelltown , Australia; 3 School of Health Studies, University of Western Ontario, 1151 Richmond Street, London, ON Canada; 4 School of Nursing, University of Victoria, HSD Building A402A, Victoria, VIC Canada; 5 Temerty Faculty of Medicine, University of Toronto, 123 Edward Street, Suite 1200, Toronto, Canada; 6 School of Social Work, McGill University, 550 Sherbrooke Ouest, Montreal, Canada; 7 College of Nursing, University of Saskatchewan, 173—1061 Central Avenue, PrinceAlbert, Canada; 8 Dalhousie University, Nova Scotia. School of Health and Human Performance, 5981 University Avenue, Room 4210F, Halifax, Canada; 9 Department of Health and Society, University of Toronto, Scarborough, 246 Bloor Street W, Toronto, Canada; 10 Alberta Health Services, 604 Main Street S, T4B 3K7 Airdrie, Alberta Canada

**Keywords:** Mental health, Adolescent, Child, Primary health care, Educational institutions, Community, Community health services

## Abstract

**Background:**

Youth mental health is a growing concern in research, practice, and policy. Practice standards, guidelines, or strategies provide an invisible infrastructure that fosters equity, quality, and safety, potentially addressing inconsistencies and more effectively attending to the mental wellness of Black youth as a particular population of concern. This scoping review aimed to address the following question: What standards exist for the delivery of mental health services to Black youth in community, primary care, and educational settings? Due to a limited initial search yield on publications about standards for the delivery of mental health services for Black youth population, our goal was then to identify and map mental health standards, recommendations, or guidelines for the delivery of mental health services using the same settings to all youth.

**Methods:**

Searches were conducted in various databases, including PubMed/MEDLINE, PsycINFO, Embase, SocINDEX, CINAHL, Gender Studies Database, Social Services Abstracts, Sociological Abstracts, Scopus, Web of Science, and Google Scholar. Screening was independently conducted by two reviewers, with disagreements resolved by a third. Information extraction was performed by two independent reviewers.

**Results:**

Out of the 2,701 screened publications, 54 were included in this scoping review. Among them, 38.9% were published between 2020 and 2023, with 40.7% originating from the United States of America, 20.4% from the United Kingdom, and 13% from Canada. Concerning the settings, 25.9% of the publications focused on primary care, 24.1% on health care services, 20.4% on educational settings, and 3.7% on the community. Additionally, 25.9% were classified as general because recommendations were applicable to various settings. Attention-deficit/hyperactivity disorder (11.1%) was the most frequently considered specific condition, followed by autism spectrum disorder (9.3%) and depression (9.3%). However, 31.5% of the included references addressed mental health in general. Only three references provided specific recommendations for the Black population.

**Conclusions:**

Recommendations, guidelines, or standards for Black youth mental health services in community, primary care, or educational settings are scarce and limited to North American countries. This scoping review emphasizes the need to consider ethnicity when developing guidelines or standards to improve racial equity and reduce disparities in access to mental health services.

**Supplementary Information:**

The online version contains supplementary material available at 10.1186/s13034-024-00800-5.

## Introduction

Youth mental health is a focus of growing concern in research, practice, and policy because the peak incidence of major mental disorders occurs in the population aged 12 to 15 years, such as anxiety, bipolar disorder, depression, eating disorders, obsessive–compulsive and psychotic disorders, schizophrenia, among others [1]. For instance, a meta-analysis estimated a worldwide- pooled prevalence of mental disorders in children and adolescent of 13.4% (CI 95% 11.3–15.9), including a prevalence of anxiety disorder, attention-deficit/hyperactivity disorder (ADHD), and depressive disorder of 6.5%, 3.4%, and 2.6%, respectively [2].

According to the Canadian Institute for Health Information, in 2020 nearly one in four hospitalizations in the population aged 5 to 24 years was for a mental health condition, with hospitalizations for these causes increasing from 21% in 2019 to 23% in 2020 [3]. In addition, children and youth aged 5 to 24 from New South Wales, Australia, showed an increase in emergency room visits for self-harm, from 371.4 per 100,000 inhabitants aged 5 to 24 in 2012 to 624.2 per 100,000 in 2020 [4]. An increase in the prevalence of mood disorders, anxiety disorders, and suicidality from 2011 and 2018 was also reported in Canadians between the ages of 12 and 24 years [5]. In addition, growth in the use of mood and anxiety medications in children and youth, from 6,071 to 7,372 dispensed medications per 100,000 inhabitants aged 5 to 24, respectively, was noted from 2016 to 2020 [3]. Also, an increase in the volume of mental health conversation services provided by the Kids Help Phone organization was observed from 2018 to 2022 [6]. Moreover, disparities related to gender, neighborhood income quintiles, level of food insecurity, and immigrant origin have been identified, with youth who identify as female, as from the least-affluent neighborhoods, with severe food insecurity, and with at least one immigrant parent being the most affected groups [3, 7–9].

Geographical differences exist in prevalence of mental health conditions among youths. For instance, the United States of America (USA) reported an autism spectrum disorder (ASD) prevalence of 3.04% [10], while a meta-analysis estimated a global ASD prevalences of 0.6% [11]. 

Regarding disparities in the frequency of diagnosed mental health conditions in Black youth, Black/non-Hispanic females in public middle and high schools from Florida, USA are reported to have a high frequency of suicide attempts in the last year (15.6%), second only to Native American females (16.5%) [12]. Moreover, a lifetime prevalence of eating disorders of 0.95% in children aged 9 and 10 was noted in the USA, with a higher prevalence in those identifying as Black compared to White [13]. Disparities in mental health treatment have also been reported in the USA related to race in adolescents with major depressive disorder, such as a longer delay in getting prescriptions in Black vs. White youth [14].

Concerning the mental health of Black youth in Canada, a study in Ontario showed that Caribbean and Bermuda immigrants and East African refugees, including youth, had a higher incidence of psychotic disorders than the general population [15]. In Montreal, African and Afro-Caribbean youth had higher scores for negative symptoms and general psychopathology scales than White French or English Canadians during first-episode psychosis [16]. Further, since the start of the COVID-19 pandemic, visible minority groups in Canada, including the Black population, have been more likely to report worse mental health and display symptoms of moderate to severe anxiety disorder compared to their White counterparts [17]. In addition, evidence shows Canadian adolescents, including Black youth, had an increase in depression and anxiety symptoms during the COVID-19 pandemic [18, 19]. Moreover, despite the universality of the Canadian health system, some systemic, practitioner-related, and personal and community-related barriers related to mental healthcare access for Black youth have been identified [20, 21].

Among the roles of providers of mental health services for children and youth, preventive care, screening, assessment, treatment, and collaborative care have been documented as necessary for improving mental health outcomes [22]. In addition, making efforts to achieve equal outcomes by addressing racial inequities in the delivery of mental health services is crucial for many population groups [23]. However, access to mental health care varies among different population groups and settings, and can be especially poor for Black youth [14, 20, 21, 24]. For instance, in the USA, it was reported that non-Hispanic Black adolescents had a lower probability of receiving mental health services [25], and for children aged 5 to 17, it was observed that disparities in mental health care use have worsened over time [26]. Additionally, in Canada, it was found that Black individuals with psychosis had a lower quality of care and a higher probability of service disengagement [27]. This suggests a gap in the quality of mental health services related to racial inequities and knowledge requirements of practitioners for supporting the mental health needs of Black youth.

In this sense, standards, including medical standards, have been considered as an “invisible infrastructure” that promotes quality and efficiency in service delivery [28]. Clinical practice guidelines have the potential to enhance healthcare quality, safety, and outcomes, as well as reduce inappropriate practice variations because they are based on a translation of scientific evidence into practical recommendations [29]. Moreover, the World Health Organization (WHO)’s guideline on person-centred and human rights-based mental health services lists inclusive practice that attends to patient diversity as one of its recommendations and calls for concentration on expanding community-based mental health services, including primary care and educational settings, to avoid hospitalizations [30]. Thus, the application of standards or guidelines related to mental health service delivery in different settings could ameliorate inconsistencies and more effectively and respectfully attend to the mental wellness of Black youth.

Amidst the escalating concern for global youth mental health [31], a notable void exists in terms of tailored standards for mental health service delivery for Black youth in community, primary care, and educational settings. This scoping review initially sought to answer the following question: What standards exist for the delivery of mental health service to Black youth? With few publications found, the review expanded to identify and map standards for the delivery of mental health service in community, primary care, and educational settings to all youth. This scarcity underscores the urgent need for culturally sensitive and equitable development of mental health services delivery standards to address disparities in access to essential services.

## Methods

### Eligibility criteria and information sources

Initially, we conducted a literature review based on three criteria: 1) focuses on mental health; 2) focuses on Black youth; and 3) relates to standards, guidelines, or recommendations for service delivery in a community, primary care, or educational setting. For the first criterion, we employed search general terms such as mental health, mental disorder, mental illness, mental wellbeing, mental wellness, emotional health, emotional wellbeing, and psychosocial wellbeing. We also utilized specific terms related to the most prevalent mental health diagnoses in youth, such as depressive disorder, dysthymia, anxiety disorder, schizophrenia, bipolar disorder, eating disorders, conduct disorder, ADHD, ASD, and idiopathic developmental intellectual disability. For the second criterion, considering the variation in the definition of children, adolescents, and youth across the countries and territories, an age cut-off was no applied. Instead, we utilized terms including adolescent, teenager, youth, young people, younger people, young adult, student, high schooler, and secondary school. In addition, terms including Black, African, Caribbean, person of color, dark-skin, and racial or ethnic minority were used. For the third criterion, we incorporated terms such as standard, care, clinical, practice, guideline, and framework (See Additional file 1, section A).

Searches were conducted in PubMed/ Ovid MEDLINE, Ovid APA PsycINFO, Ovid Embase, EBSCOhost SocINDEX, EBSCOhost CINAHL, EBSCOhost Gender Studies Database, ProQuest Social Services Abstracts, ProQuest Sociological Abstracts, Scopus, and Web of Science Core Collection via Clarivate (See Additional file 1), without time or language restrictions. Additionally, searches were conducted in Google Scholar to include grey literature. Only 32 references met at least two of the three criteria when titles and abstracts were screened. As a result, we decided to extend the search in the same databases omitting terms related to the Black population (Black, African, Caribbean, person of color, dark-skin, and racial or ethnic minority), while keeping the focus on guidelines for delivery of mental health services in community, primary care, and educational settings to youth (See Additional file 1, section B). The initial searches were executed on September 12, 2023, and the extended searches were executed between October 3 and October 23, 2023. The screening of titles and abstracts was conducted independently by two of three reviewers, with disagreements resolved by a third (RM-V, OU, VA). Eligible full-text references were then screened independently by two reviewers (RM-V, OU). When more than one version of the same guideline was noted, the updated version was included and any older versions excluded. In addition, some specific manuscripts identified through manual search during the review of the references of selected publications were included. We excluded publications that focused on the wrong setting (secondary health care or in-hospital treatment) or wrong population (guidelines were only for preschoolers or adults), or were commentaries about guidelines or abstracts from meetings, among others. Manuscripts focusing on a specific intervention, such as clinical trials, protocols, or meta-analyses, were also excluded. The screening was conducted using Covidence systematic review software, Veritas Health Innovation, Melbourne, Australia.

### Data extraction

Data extraction was done on Excel spreadsheet (See Additional file 2) including the: source of the reference; year of publication; purpose of the guidelines, recommendations, or standards; methodology used for the elaboration of recommendations; source country for the guidelines, recommendations, or standards elaborated; target population (children, adolescents, youth, scholars, specific age groups, among others); care setting (primary care, school, community, health care services when the health care setting was not specified, and general setting when recommendations were made for different settings); specific condition (e.g., ADHD, depression, suicide, general mental health); recommendations; and if specific recommendations for the Black population were made (Yes/No). The agency funding the study was also extracted. The information extraction was done by two independent reviewers (RM-V, OU), and in the case of discrepancies, a reviewer (RM-V) revisited the article to address and resolve them.

### Synthesis of results

The characteristics of the selected references were described using absolute frequencies and percentages. The flow diagram was adapted from Covidence, and other figures were created using Stata Software 16.1. Critical appraisal of individual sources was not conducted because variety of methodologies used in the included publications, and it was outside the scope of the review objective. This report adheres the Preferred Reporting Items for Systematic reviews and Meta-Analyses extension for Scoping Reviews (PRISMA-ScR) Checklist.

## Results

**Fig. 1:**
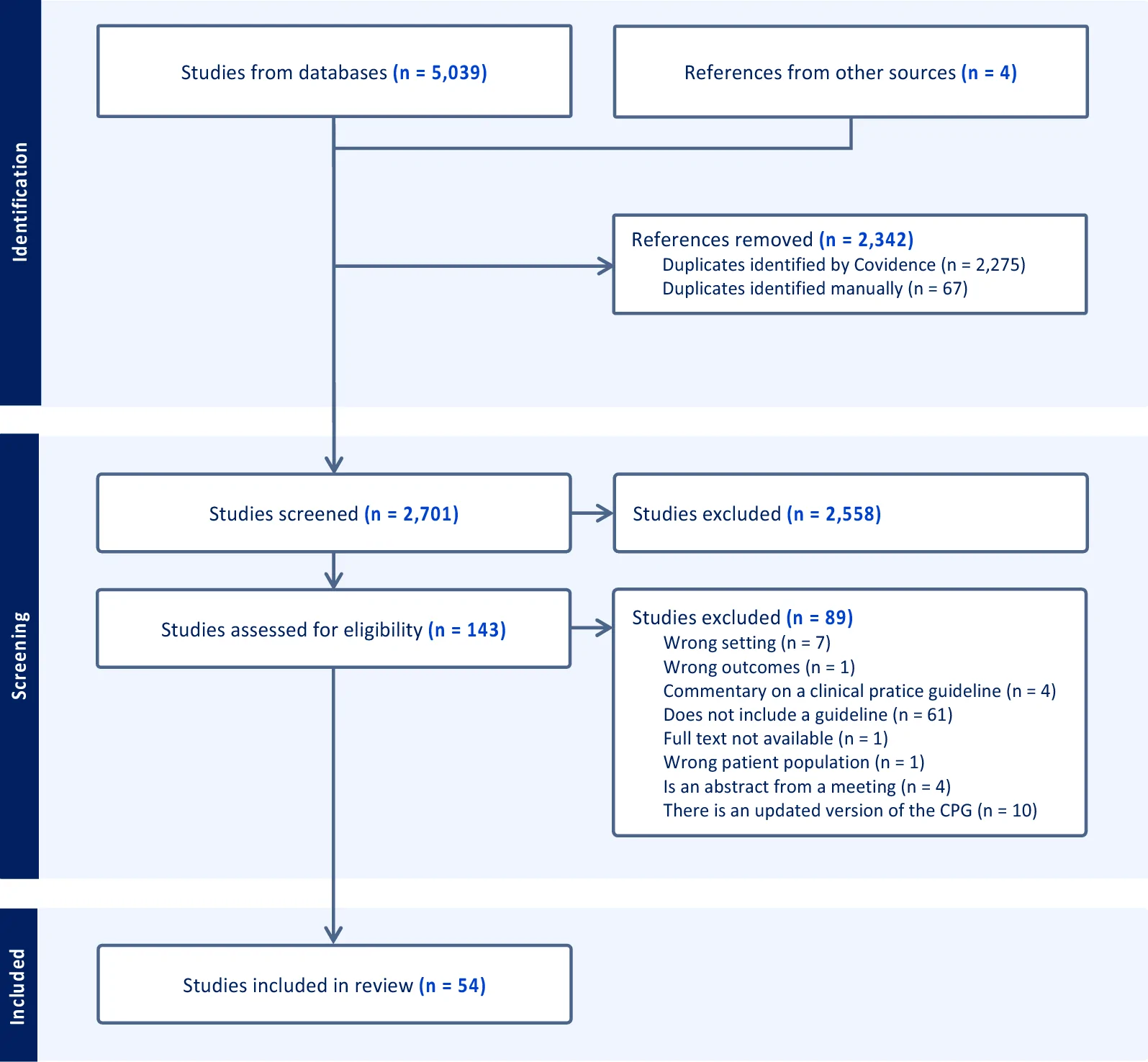
Flow diagram

In total, 5,043 publications were identified after the search was extended, omitting terms related to the Black population while keeping the focus on guidelines, recommendations, or standards for mental health of youth, of which 2,342 (46.4%) were duplicates and removed. The remaining 2,701 publications were screened by title and abstract, resulting in the exclusion of 2,558 articles. A total of 143 (5.3%) were deemed eligible for full-text review, with only 54 publications meeting our inclusion criteria and retained for data extraction (See Additional file 2). The primary reason for exclusion was that the reference did not constitute a guideline, recommendation, or standard (See Additional file 3), followed by instances of an outdated version of the guideline (Fig. 1).

**Fig. 2:**
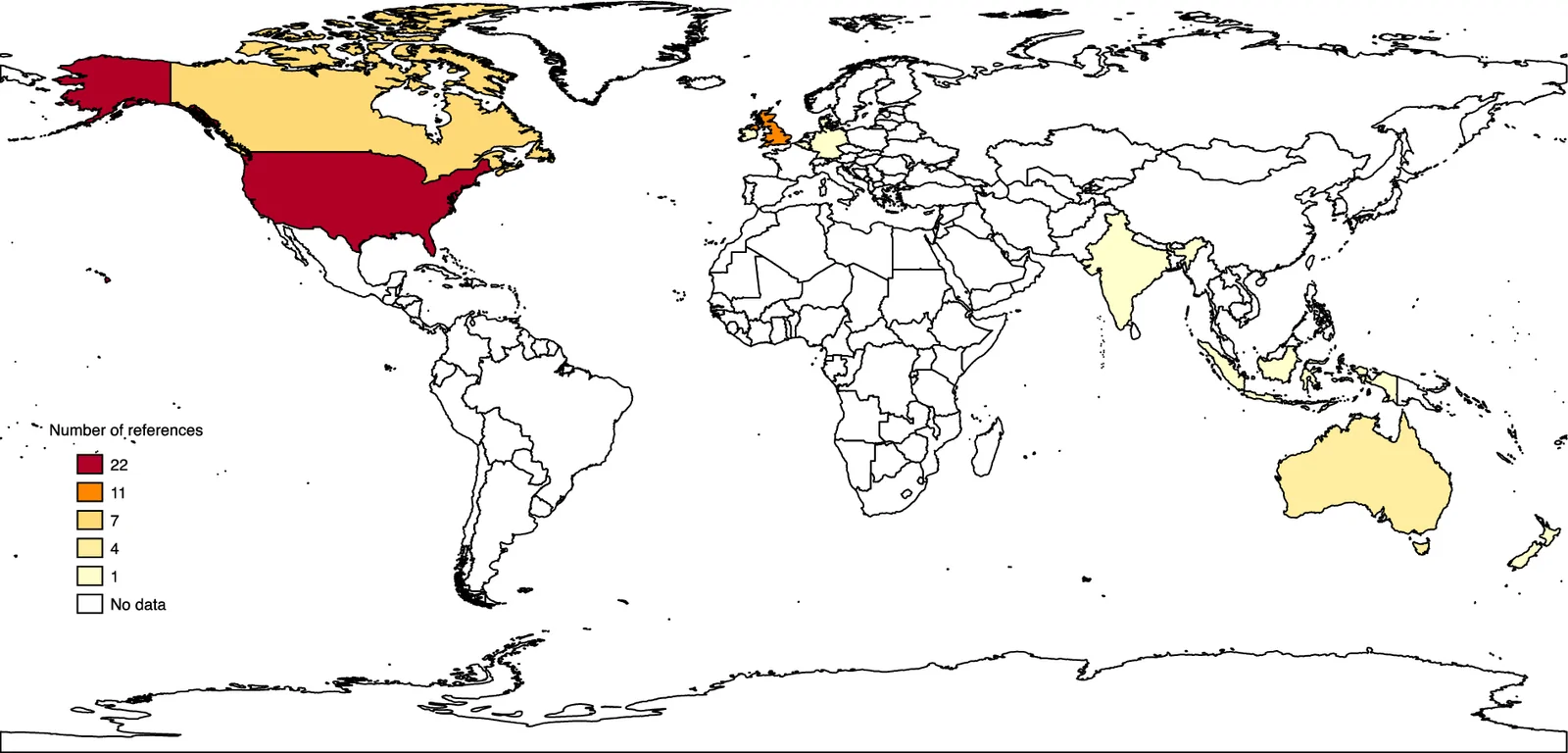
Distribution of the included references by country of origin

Of the included references, 11.1% (n = 6) were published from 2005 to 2009, 22.2% (n = 12) from 2010 to 2014, 27.8% (n = 15) from 2015 to 2019, and 38.9% (n = 21) from 2020 to 2023. Except for one [32], all selected publications were in the English language. Of the selected references, 52 (96.3%) were from 11 countries (Australia, Belgium, Canada, Denmark, Germany, India, Indonesia, Ireland, New Zealand, the United Kingdom [UK], and the USA). Most publications were from the USA (n = 22, 40.7%, including two guidelines about depression in collaboration with Canadian experts), followed by the UK (n = 11, 20.4%), Canada (n = 7, 13.0%), and Australia (n = 4, 7.4%) (Fig. 2). Additionally, three publications were from international groups: the first about general mental health from the WHO [33]; the second about anxiety, depression, obsessive–compulsive disorder, and post-traumatic stress disorder (PTSD) from an International Consortium [34]; and the third about eating disorders from the European Academy of Paediatrics [35] (See Additional file 2).

Regarding the target population for the guidelines, recommendations, or standards, 37% (n = 20) of the publications specified an age cut-off, with 25 years being the highest age considered for defining youth. In addition, 13% (n = 7) of the publications included youth and adults [32, 36–41]. In terms of setting, the largest percentage of the publications (n = 14, 25.9%) reflected primary care, followed by health care services (n = 13, 24.1%), education settings (n = 11, 20.4%), specifically schools (n = 9), collegiate level (n = 1), and tertiary education (n = 1), and community settings (n = 2, 3.7%). In addition, 25.9% (n = 14) were classified as general because recommendations were made for different settings, including health services, educational settings, and/or community (See Additional file 2).

**Fig. 3:**
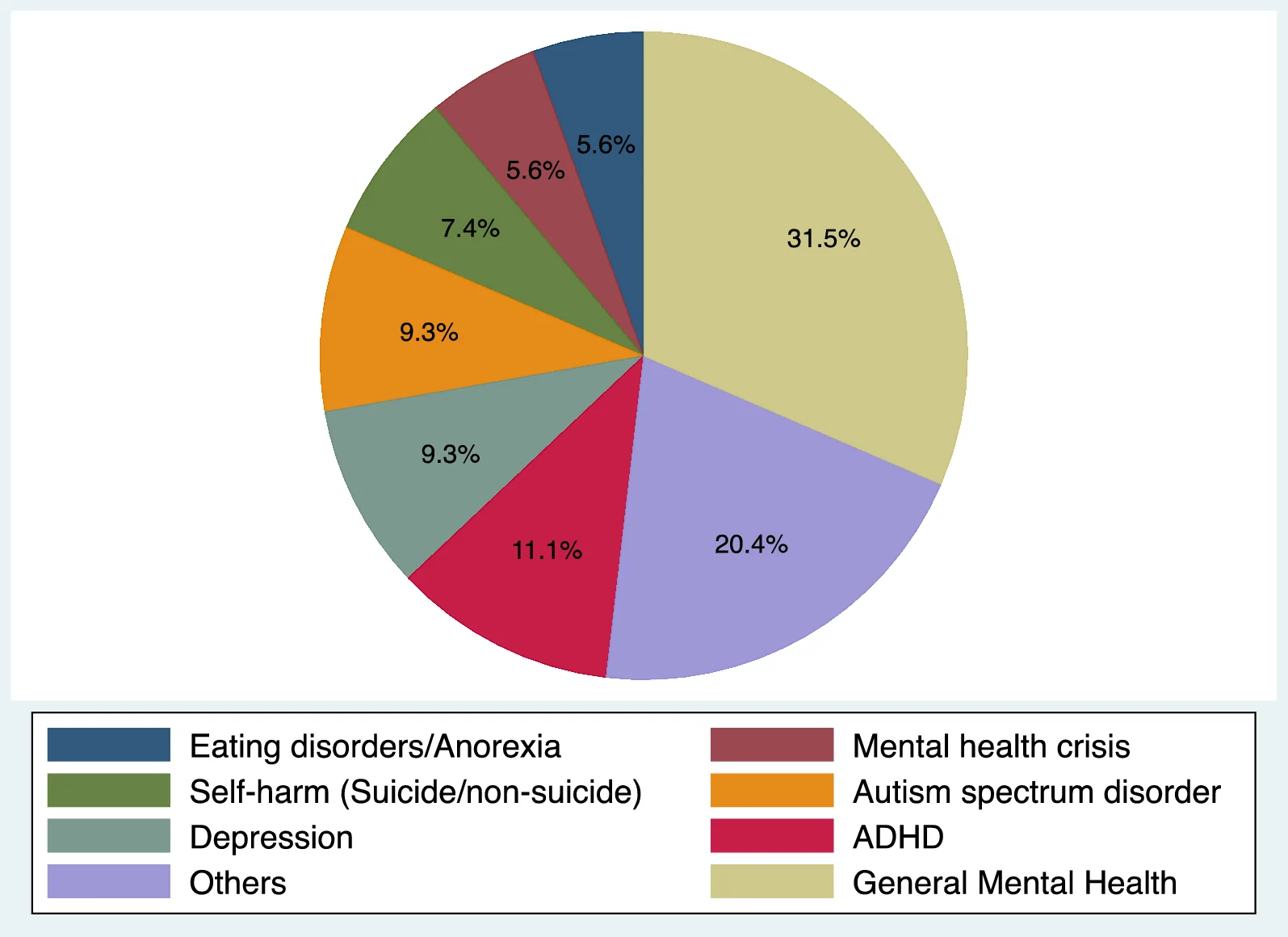
Distribution of the included references by mental health condition(s) considered

The guidelines, recommendations, or standards covered a variety of mental health conditions, with the most frequent being ADHD (n = 6, 11.1%) [42–47] followed by ASD (n = 5, 9.3%) [32, 48–51] and depression (n = 5, 9.3%) [34, 52–55] (Fig. 3). One publication about depression also discussed suicide [54], and another considered anxiety, depression, obsessive–compulsive disorder, and PTSD [34]. In addition, four (7.4%) references focused on self-harm (suicide or non-suicidal self-injury) [56–59], three on eating disorders/anorexia (5.6%) [35, 40, 60], and three on mental health crises (5.6%) [41, 61, 62]. Moreover, two gave recommendations regarding anxiety [38, 63] and two gave guidelines for maladaptive aggression [64, 65]. Seven publications focused on other specific conditions, namely bipolar disorder [39], learning difficulties/disabilities experiencing mental health problems/challenging behaviour [66], PTSD [67], specific learning disorder [68], gender dysphoria [69], traumatic events [70], and violence and aggression [36]. The remaining papers (n = 17, 31.5%) were about mental health in general (Fig. 3).

### ADHD: Attention-deficit/hyperactivity disorder

In terms of the methodology used for developing guidelines or recommendations, 11.1% (n = 6) of the publications were reviews of the literature [35, 47, 51, 60, 68, 71], 11.1% (n = 6) used Delphi methodology [34, 59, 61, 72–74], 11.1% (n = 6) used National Institute for Health and Care Excellence (NICE) methodology [36, 39, 48, 49, 55, 66], and 11.1% (n = 6) combined reviews of the literature with expert consensus [43, 44, 52, 53, 67, 75]. Also, 14.8% (n = 8) included patients or family members during the development process [42, 64, 65, 69, 76–79], 5.6% (n = 3) were developed by the US Preventive Services Task Force (USPSTF) using their methodology [40, 54, 63], and 3.7% (n = 2) applied a combination of USPSTF methodology with expert groups [38, 80]. In addition, the Task Force on Community Preventive Services from the USA [70], the Association of the Scientific Medical Societies in Germany (AWMF) [32], and the WHO [33] each developed a set of guidelines using their own procedures (5.6%). Several (n = 9, 16.7%) of the references applied different approaches [41, 45, 46, 56, 58, 62, 81–83], while for 9.3% (n = 5) the methodology could not be determined [37, 50, 57, 84, 85] (See Additional file 2).

Only three references, all from USA (n = 2) and Canada (n = 1), had specific recommendations for Black populations, specifically Black youth [71], young Black men [85], and Black communities in Western countries [37]. The first [71] focused on practice recommendations for addressing the gap between evidence about culturally competent mental health therapeutic interventions and routine clinical practice. Among the recommendations were: two related to attitudes and beliefs such as talking to families about their prior experiences with therapy and the system and discussing limits to confidentiality vs. privacy; four related to addressing access barriers, such as providing bus passes or other alternatives for transportation, making reminder calls, helping arrange or providing childcare, and offering evening and weekend hours or telehealth; and six that considered cultural strengths and risks, using role play responses to racism and discrimination in sessions, processing racial stressors to cognitively reframe and regulate emotions, assigning homework to facilitate racial pride messages and practices, harnessing Africentric notions of verve and rhythm in therapy rooms, incorporating prayer or meditation into relaxation and coping, and utilizing extended family members and fictive kin in treatment (See Additional file 2).

The second reference [85] made four policy recommendations for innovative policy and programming changes to better meet the psychosocial needs of young men of color: 1) acknowledge and work to directly address and remedy the legacy of structural racism that continues to impact the lives of young men of color, 2) be as flexible as possible and continuously evolve to meet the changing needs of participants, 3) use trauma-informed practices to foster collaboration and enhance feelings of safety and agency, and 4) create a participant-to-staff pipeline by providing professional experiences for participants who may then be hired as staff. The third reference [37] featured guidelines to provide anti-racist mental health care, addressing four main concerns: 1) an awareness of racial issues (eight recommendations), 2) an assessment adapted to the real needs of Black individuals (nine recommendations), 3) a humanistic approach to medication (two recommendations), and 4) a treatment approach that addresses the real needs and issues related to racism experienced by Black individuals (eight recommendations) (See Additional file 2).

Regarding other specific youth populations, two publications focused on Indigenous populations, namely one on Aboriginal and Islander adolescents from Australia [61] and the other on American Indian/Alaska Native adolescents from the USA [76]. In addition, one reference focused on girls/women aged 13 years or older [38], and two focused on student athletes [75, 81].

## Discussion

The combined insights from Jones et al. [71], Cénat [37], and Connolly et al. [85] shed light on comprehensive strategies and recommendations to enhance mental health outcomes for Black youth and young men of color. Collectively, these sources emphasize the critical importance of culturally competent and anti-racist approaches within therapeutic interventions and mental health programs. Jones et al.'s [71] emphasis on open communication, understanding prior therapy experiences, and addressing practical barriers such as transportation aligns with the broader theme of recognizing and overcoming systemic challenges. The incorporation of culturally relevant elements, such as Africentric notions and spiritual practices, reflects an awareness of the need for culturally sensitive therapeutic strategies. Cénat's [37] guidelines provide a deeper understanding of anti-racist mental health care, emphasizing the significance of self-examination among clinicians to be aware of their biases and privileges. The guidelines stress the importance of culturally adapted assessments, recognition of diverse cultural backgrounds, and a humanistic approach to medication. The approach to medication is particularly noteworthy, advocating for transparency, communication, and understanding of the client's perspective to rebuild trust in mental health services. In addition, applying the recommended humanistic approach to medication, which includes prescribing medication only if there are no other alternatives and considering that, although prescribing is a quick and easy response, people have other needs, would support increasing the availability of mental health service in other settings such as schools or community centers.

Building upon these individual-focused recommendations, Connolly et al. [85] offer policy-level suggestions to address mental health equity for young men of color. Acknowledging the legacy of structural racism and fostering flexibility in mental health programs demonstrates a systemic awareness. The emphasis on trauma-informed practices and creating opportunities for participants to transition into staff roles contributes to a holistic approach that considers both individual and systemic factors.

Jones et al. [71] and Connolly et al. [85] recommend that programs should be flexible and adaptable in terms of place, time, programming, and staffing. These recommendations are essential for developing programming that not only considers the psychosocial needs of Black youth but also overcomes the barriers in accessing mental healthcare that have been described for this population [20]. For instance, systemic barriers such as wait times for primary care and geographical barriers due to low availability of mental health services in low-income communities, and practitioner-related barriers such as racism and discrimination, could be overcome if theses recommendation are put into practice.

Thus, the results suggest a multi-faceted approach to improving mental health outcomes for Black youth and young men of color, encompassing individual therapeutic practices, systemic changes, and policy-level interventions. This integrated perspective underscores the need for a comprehensive and culturally competent framework to address the diverse challenges faced by these populations in the realm of mental health.

The limited number of publications about guidelines, standards, or recommendations for the delivery of mental health services in Black youth in community, primary care, and educational settings necessitated the expansion of the terms of this scoping review and extension of the search. The extended search allowed us to identify 54 articles that were mainly published in USA and UK after 2010, the main scope of which was health care settings. About a third of the publications focused on general mental health, with ADHD the most frequently considered specific condition. Considerable variability was noted in terms of methodology, with Delphi and NICE methodologies being equally used, similar to the literature review alone or with expert consensus. Furthermore, most of the selected references considered the youth population in general, and only eight considered a target group such as Indigenous or Black youths, females, or student athletes.

Although the evidence indicates Black youth are more likely to report mental health conditions and have poor social determinants of health, such as poverty and inadequate housing, and the WHO has advised regarding the development of inclusive mental health services to ensure available, accessible, and culturally acceptable services [30], only three publications offered recommendations or guidelines for the delivery of Black youth population mental health services in community, primary care, or educational settings. The three that did were also all based in North American countries [37, 71, 85]. These results highlight the need to consider ethnicity when a guideline or standard is being developed to improve racial equity and diminish disparities in access to youth mental health services [23]. Moreover, the UK and Australia are the other two high-income countries with a significant number of selected publications, but no guidelines for Black youth were developed in these countries even though both have Black population. Particularly in Australia, the deficiency in considering race in data collection has previously been exposed as a factor that threatens the achievement of health equity [86].

Notably, around one-fifth of publications offering guidelines, standards, or recommendations focused on educational settings [47, 51, 56, 57, 59, 68, 72, 73, 75, 81, 83, 84]. Because youth spend much of their daily time in schools, these settings are recognized as an essential avenue where mental health prevention, promotion, screening, interventions, and referrals can be done [87, 88]. Educational settings have many advantages such as simple access to a large number of youths, the possibility of providing a range of proven mental health interventions, and links with primary health providers, among others [88].

On the other hand, a wide variety of mental health conditions were considered in the selected references. Consistent with the prevalence of mental health conditions reported in the international literature [3, 4, 10], guidelines or recommendations found in this scoping review largely related to ADHD [42–47], depression [34, 52–55], and self-harm [56–59]. Notably, an equal number of publications focused on depression and ASD [32, 48–51] even though ASD is less prevalent [10, 11].

Among the limitations of this scoping review, we point out that all but one publication were in English, even though no language filters were used. This likely occurred because the keywords used in all searches were in English and might not have been sensitive enough to detect literature in other languages. This language limitation also could be related to few references from middle- or low-income countries. Another limitation was that the quality of guidelines or recommendations was not evaluated.

Ultimately, the three manuscripts providing recommendations or guidelines for delivering mental health services to the Black youth population in community, primary care, educational settings originated from high-income North American countries, thereby limiting generalizability of these recommendations to other regions. Nevertheless, this scoping review has enabled us to identify this gap, making a crucial first step in addressing racial inequities and supporting the mental health needs of Black youth globally. Given the nascent state of mental health research in the Black population, we advocate for prioritizing the development of both quantitative and qualitative research dedicated to the Black population and other minorities in local agendas. Furthermore, we advocate for intersectoral collaboration, involving stakeholders such as academics, researchers, health providers, and public sector, in the formulation of standards aimed at mitigating racial inequities in delivering mental health services.

## Conclusions

Although the evidence indicates Black youth are more likely to report mental health conditions and barriers to accessing mental healthcare, current publications featuring recommendations, guidelines, or standards for Black youth mental health service delivery in community, primary care, or educational settings are scarce and limited to USA and Canada. The extended search carried out in this scoping review allowed us to identify 54 publications about mental health recommendations, guidelines, or standards for youth, most of which were from the USA or UK and focused on general mental health, ADHD, depression, and/or ASD.

## Supplementary Information

Additional file 1

Additional file 2

Additional file 3

## Abbreviations

ADHD: Attention-deficit/hyperactivity disorder.
ASD: Autism spectrum disorder.
AWMF: Association of the Scientific Medical Societies in Germany.
NICE: National Institute for Health and Care Excellence.
PTSD: Post-traumatic stress disorder.
UK: United Kingdom.
USA: United States of America.
USPSTF: US Preventive Services Task Force.
WHO: World Health Organization
