# Supplementary material for: Scoping review on mental health standards for Black youth: identifying gaps and promoting equity in community, primary care, and educational settings
Source: Child Adolesc Psychiatry Ment Health. 2024 Sep 9;18:113. doi: 10.1186/s13034-024-00800-5 (PMC11385802; doi:10.1186/s13034-024-00800-5)
Supplement: Supplementary file 1 — Additional file 1 [file 13034_2024_800_MOESM1_ESM.docx]

**Scoping review on global mental health standards for Black youth: Identifying gaps and promoting equity in community, primary care, and educational settings**

**Supplementary material**

1. **Search strategy in Ovid Embase (1974 to September 11, 2023** **// Last searched September 12, 2023).**

| **#** | **Terms** | **References** |
| --- | --- | --- |
| 1 | exp mental health/ or exp mental disease/ or exp mental health care/ | 2811679 |
| 2 | ("mental health" or "mental disorder*" or "mental illness*" or "mental wellbeing" or "mental well-being" or "mental wellness" or "emotional health" or "emotional wellbeing" or "emotional well-being" or "emotional wellness" or "psychosocial wellbeing" or "psychosocial well-being" or "psychological wellbeing" or "psychological well-being" or "depressive disorder*" dysthymia or "anxiety disorder*" or schizophrenia or "bipolar disorder" or "eating disorders" or "conduct disorder" or "attention deficit hyperactivity disorder" or adhd or "autism spectrum disorder*" or asd or "idiopathic developmental intellectual disability").ti,ab. | 708174 |
| 3 | 1 or 2 | 2921282 |
| 4 | adolescent/ or young adult/ or juvenile/ | 2148914 |
| 5 | (Adolescen* or teen* or youth? or "young people" or "younger people" or "young adult?" or "young women" or "young men" or student? or "high schooler?" or "secondary school").ti,ab. | 1162514 |
| 6 | 4 or 5 | 275423 |
| 7 | exp Black person/ or exp African/ | 180963 |
| 8 | (black* or african* or caribbean or afro* or "person of colo?r" or "people of colo?r" or colo?red or "dark-skin*" or BIPOC or ((racial or ethnic) adj2 minorit*)).ti,ab. | 595266 |
| 9 | 7 or 8 | 643662 |
| 10 | practice guideline/ or clinical protocol/ or professional standard/ | 707278 |
| 11 | ((standard* adj2 (care or clinical or practice)) or (guideline* adj2 (care or clinical or practice)) or (framework* adj2 (care or clinical or practice))).ti,ab. | 273520 |
| 12 | 10 or 11 | 910700 |
| 13 | 3 and 6 and 9 and 12 | **366** |

1. **Extended search strategy in Ovid Embase (1974 to October 22, 2023 // Last searched October 23, 2023).**

| **#** | **Terms** | **References** |
| --- | --- | --- |
| 1 | (standard* or guideline* or framework* or recommendation* or strategy*).m_titl. | 492132 |
| 2 | (adolescen* or teen* or youth or "young people" or "younger people" or "young adult*" or "young women" or "young men" or student* or "high schooler" or "secondary school").m_titl. | 532513 |
| 3 | ("mental health" or "mental disorder*" or "mental illness*" or "mental wellbeing" or "mental well-being" or "mental wellness" or "emotional health" or "emotional wellbeing" or "emotional well-being" or "emotional wellness" or "psychosocial wellbeing" or "psychosocial well-being" or "psychological wellbeing" or "psychological well-being" or "depressive disorder* dysthymia" or "anxiety disorder*" or schizophrenia or "bipolar disorder" or "eating disorders" or "conduct disorder" or "attention deficit hyperactivity disorder" or adhd or "autism spectrum disorder*" or asd or "idiopathic developmental intellectual disability").mp. [mp=title, abstract, heading word, drug trade name, original title, device manufacturer, drug manufacturer, device trade name, keyword heading word, floating subheading word, candidate term word] | 984271 |
| 4 | 1 and 2 and 3 | **826** |
